# PTPN1 Regulation via YBX1-PTBP1 Interaction Promotes Fibroblast Activation and Fibrotic Remodeling in the Lung

**DOI:** 10.7150/ijbs.123875

**Published:** 2026-01-30

**Authors:** Huibing Liu, Cong Xia, Yingying Zhang, Yulong Gan, Lianhui Cheng, Xuqian Wang, Airu Chang, Wenyu Zhao, Bin Li, Yaxuan Wang, Yajun Li, Ivan Rosas, Juntang Yang, Guoying Yu, Lan Wang

**Affiliations:** 1 State Key Laboratory of Cell Differentiation and Regulation, Henan International Joint Laboratory of Pulmonary Fibrosis, Henan Center for Outstanding Overseas Scientists of Organ Fibrosis, Institute of Biomedicine College of Life Science, Henan Normal University, Xinxiang 453007, China.; 2 Department of Medicine Pulmonary, Critical Care and Sleep Medicine, Baylor College of Medicine, USA.

**Keywords:** YBX1, YBX1-PTBP1 interaction, PTPN1, fibroblast activation

## Abstract

Idiopathic pulmonary fibrosis (IPF) is a chronic, progressive interstitial pneumonia of unknown etiology. Its pathogenesis involves complex multicellular interactions and signaling pathways, with fibroblast-to-myofibroblast transition (FMT) being critical for fibrogenesis. Although the transcription factor Y-box binding protein 1 (YBX1) regulates processes such as cell proliferation, transcription, translation, and DNA repair, its role in IPF remains undefined. Here, we demonstrate that YBX1 overexpression significantly promotes transforming growth factor-β1 (TGF-β1)-induced pulmonary FMT, leading to substantially increased extracellular matrix (ECM) deposition in primary human (PHLFs) and mouse (PMLFs) lung fibroblasts. Conversely, YBX1 inhibition markedly suppresses TGF-β1-driven aberrant fibroblast migration and activation. Mechanistically, YBX1 interacts with polypyrimidine tract-binding protein 1 (PTBP1) and binds to the protein tyrosine phosphatase nonreceptor type 1 (PTPN1) promoter to transcriptionally regulate PTPN1, thereby driving FMT. *In vivo*, intratracheal delivery of Ybx1-targeting shRNA via adeno-associated virus (AAV) robustly attenuates ECM deposition, hydroxyproline content, and fibrotic marker expression in a bleomycin (BLM)-induced murine fibrosis model. Our findings identify YBX1 as a promoter of lung FMT via the PTBP1/PTPN1 axis, offering mechanistic insights for the development of YBX1-targeted therapeutic strategies for IPF.

## Introduction

IPF is a chronic and progressive fibrotic lung disease characterized by scarring of the lungs, leading to dyspnea and irreversible loss of lung function. IPF typically manifests in adults over 60 years of age, and the prognosis is poor, with a median survival of only 3-5 years after diagnosis 1, 2. Although the recent introduction of two antifibrotic drugs, pirfenidone and nintedanib, has resulted in a significant reduction in lung function decline 3, 4, the etiology and physiological mechanisms of IPF remain incompletely understood.

The activation of fibroblasts has become a core link in the pathogenesis of IPF 5-7. At a deeper level, genetic factors and epigenetic regulation also play important roles in the activation of myofibroblasts. Variations or abnormal expression regulation of certain genes may lead to increased sensitivity of cells to stimulating factors, thereby promoting the activation of myofibroblasts 8, 9. RNA-binding proteins (RBPs) are central to this process. They are essential binding partners 10 that dynamically associate with intracellular RNAs to regulate their metabolism. By binding to target transcripts, RBPs orchestrate critical posttranscriptional gene expression mechanisms 11 and are involved in various aspects of RNA metabolism, including RNA splicing, sequence editing, transport, maintenance of RNA stability and degradation, and intracellular localization and translation control 12.

YBX1 is a multifunctional RNA-binding protein consisting of three main domains: a variable amino terminus, a highly conserved nucleic acid-binding domain (CSD), and a carboxyl terminus. Previous studies have demonstrated that it acts as a transcription factor capable of regulating the expression of PTPN1 13, YBX1 specifically binds the 5'-CCAAT-3' motif in gene promoters and enhancers to regulate their transcriptional activity and influences cellular invasion and migration. YBX1 is involved in a variety of key cellular functions, including DNA repair, transcriptional and translational regulation, alternative mRNA splicing, and mRNA packaging 14. The cellular activity of YBX1 is involved in processes such as cell proliferation and differentiation, apoptosis, stress response, and malignant cell transformation 15. YBX1 also participates in fibrotic effects through the TGF-β1 pathway and the production of inflammatory factors by mediating the epithelial-mesenchymal transition, regulating collagen expression and other mechanisms 16, 17. Therefore, this study aimed to elucidate the function of YBX1 in pulmonary fibrosis and explore its potential as a novel therapeutic target for this devastating disease.

## Results

### YBX1 expression is significantly elevated in IPF and fibrotic mouse lungs

**Figure 1:**
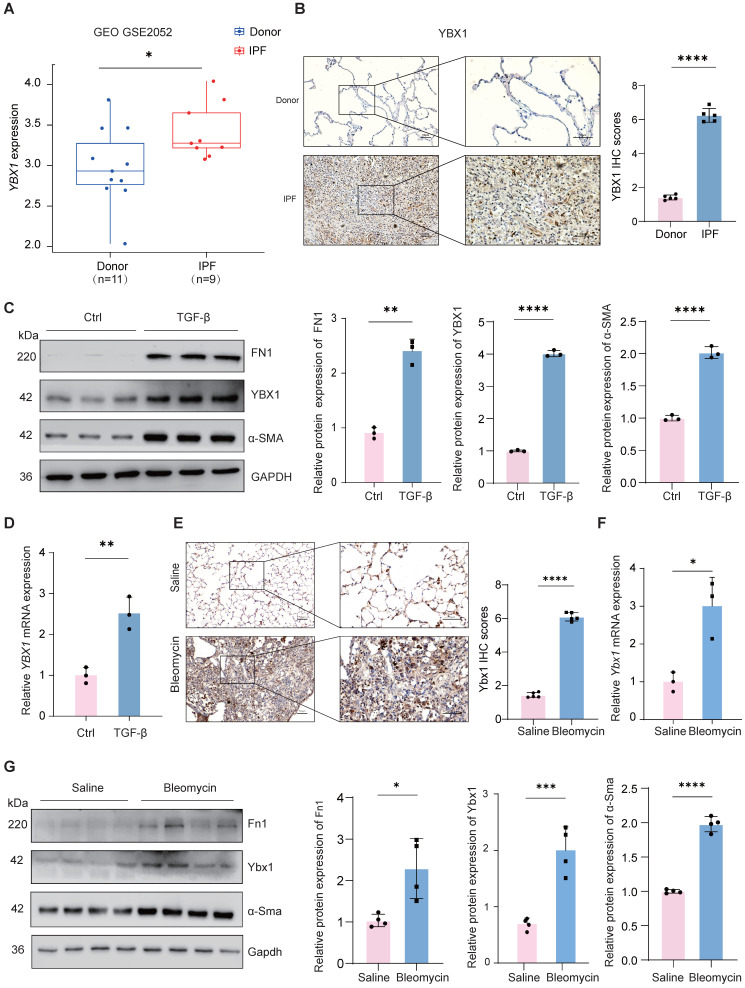
** YBX1 is upregulated in fibrotic lungs of human and mice. A** Expression of YBX1 in IPF patients was analyzed using data from GSE2052. **B** Representative images of YBX1 IHC staining in lung sections from IPF patients and control subjects (n=5). **C** Western blot analysis of FN1, α-SMA and YBX1 protein levels in TGF-β1-induced primary human lung fibroblasts (n=3). **D** qRT-PCR analysis of *YBX1* mRNA levels in TGF-β1-induced primary human lung fibroblasts (n=3).** E** Representative images of Ybx1 IHC staining in lung sections from BLM-induced and saline-treated control (n=5). **F** qRT-PCR analysis for the transcriptional changes of Ybx1 expression in mice treated with saline or BLM (n=3). **G** Western blot was used to analyze the changes in the levels of Fn1, α-Sma and Ybx1 protein in mice treated with saline or BLM (n=4). Data are shown as the mean ± SD. Significance was assessed using 2-tailed Student's t-test. *P<0.05, **P<0.01, ***P<0.001, ****P<0.0001

To investigate the overall expression of YBX1 in IPF, we first analyzed a public microarray dataset (GEO: GSE2052) and found that compared with those in healthy donors, the *YBX1* mRNA levels in lung tissue from patients with IPF were significantly upregulated (Fig. 1A). Histological staining of lung tissues from patients with IPF also revealed a marked increase in YBX1 expression compared with that in lung tissues from controls (Fig. 1B). Furthermore, we induced fibroblast activation *in vitro* by treating PHLFs with TGF-β1, a key profibrotic cytokine, and observed that TGF-β1 significantly induced the expression of the fibrotic markers α-smooth muscle actin (α-SMA) and fibronectin (FN1). Compared with those in the control group, the protein and transcriptional levels of YBX1 were markedly increased in the TGF-β1-treated cells (Fig. 1C, D). Similarly, Ybx1 expression was significantly elevated in lung tissue sections from a BLM-treated mouse model of pulmonary fibrosis (Fig. 1E). Consistent with these findings, BLM administration in mice led to significant upregulation of Ybx1 expression at both the mRNA and protein levels in lung tissues, accompanied by elevated expression of the fibrotic markers α-smooth muscle actin (α-Sma) and fibronectin (Fn1) (Fig. 1F). Together, these data demonstrate that YBX1 expression is elevated in fibrotic lung tissue and lung fibroblast models, highlighting its potential involvement in the initiation and progression of pulmonary fibrosis.

### Overexpression of YBX1 enhances TGF-β1-induced lung FMT

**Figure 2:**
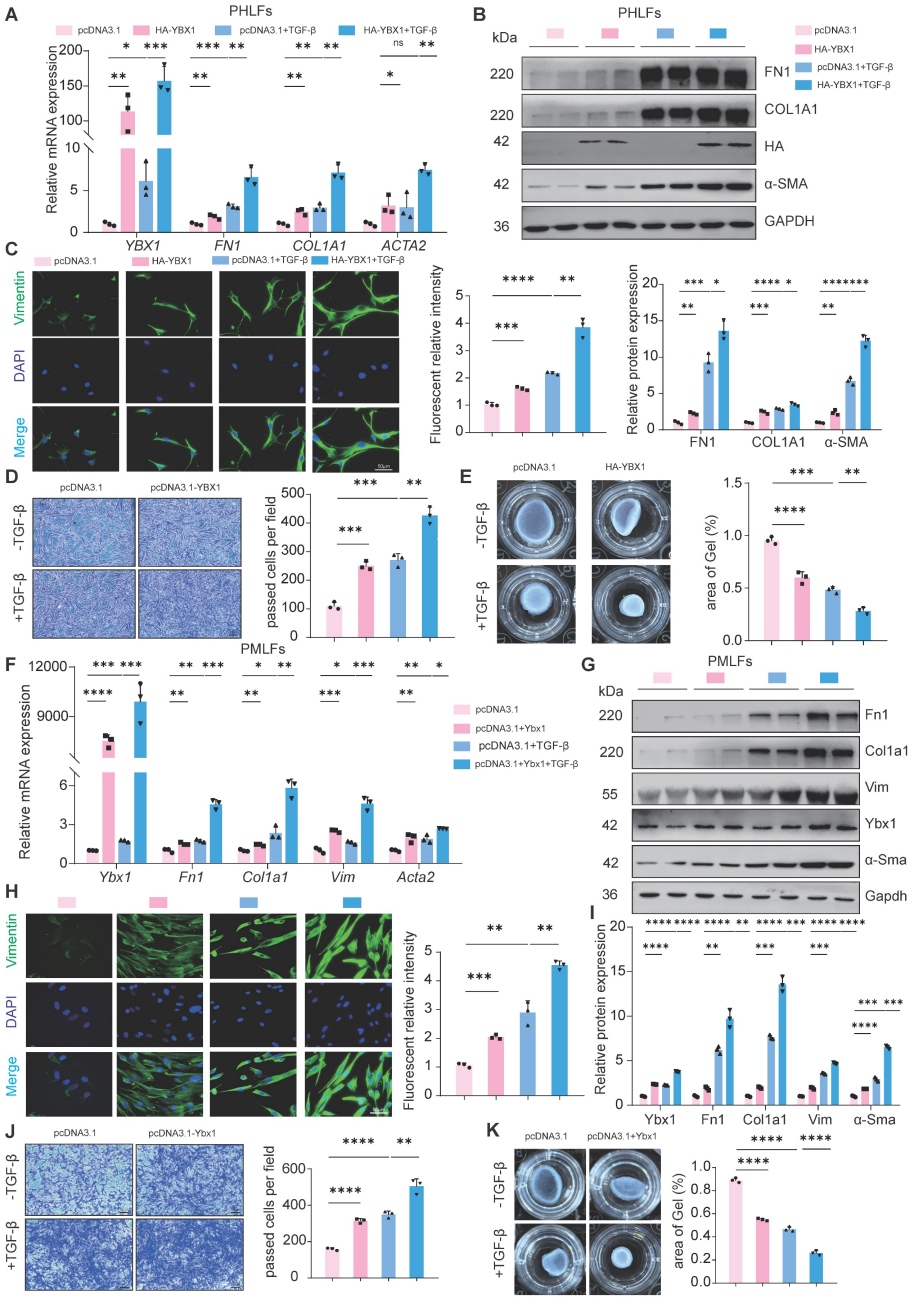
** YBX1 overexpression enhances fibrotic gene expression and TGF-β1-induced fibroblast activation in PHLFs and PMLFs. A**,** B** qRT-PCR(**A**) and Western blot(**B**) were used to analyze the expression of FN1, COL1A1 and α-SMA induced by TGF-β1 after YBX1 overexpression (n=3). **C** Immunofluorescence analysis revealed that YBX1 expression significantly heightened the expression of vimentin in PHLFs. The nuclei were counterstained with DAPI. Bar = 20 μm, (n=3). **D** YBX1 is critical for cell migration in a transwell assay. Bar = 100 μm, (n=3). **E** The fibroblast-induced CGC assay demonstrated that YBX1 overexpression in PHLFs promoted fibroblast contractility (n=3). **F** qRT-PCR analysis validated the mRNA levels of *Ybx1, Fn1, Col1a1* and *Acta2* in PMLFs. Bars represent mean values of mRNA levels normalized to *Gapdh* mRNA (n=3). **G** Western blot assay showing the protein level of Fn1, Col1a1, Vimentin, Ybx1, α-Sma and Gapdh in PMLFs (n=3). **H** Immunofluorescence analysis revealed that Ybx1 expression significantly heightened the expression of vimentin in PMLFs. The nuclei were counterstained with DAPI. Bar = 20 μm, (n=3). **I** Transwell demonstrated the effect of Ybx1 overexpression on PMLFs migration after 36h. Scale bar = 100 μm, (n=3). **J** The fibroblast-induced CGC assay demonstrated that Ybx1 overexpression in PMLFs inhibited fibroblast contractility (n=3). Data are shown as the mean ± SD. Significance was assessed using 2-tailed Student's t-test. *P<0.05, **P<0.01, ***P<0.001, ****P<0.0001

To determine the effect of YBX1 on lung FMT, we overexpressed YBX1 in PHLFs and found that it sufficiently increased the expression of TGF-β1-induced profibrotic genes, including type I collagen (COL1A1), FN1 and α-SMA, at both the mRNA (Fig. 2A) and protein levels (Fig. 2B). Immunofluorescence staining revealed that YBX1 overexpression significantly increased TGF-β1-induced vimentin expression (Fig. 2C), which was accompanied by increased TGF-β1-driven migration (Fig. 2D) and collagen gel contraction (CGC) capacity (Fig. 2E) in PHLFs. Parallel experiments in PMLFs recapitulated these findings, demonstrating that Ybx1 overexpression amplifies TGF-β1-induced pro-fibrotic gene and protein expression (Fig. 2F-H), which is accompanied by enhanced TGF-β1-driven migration (Fig. 2I), and CGC capacity (Fig. 2J). In summary, overexpression of YBX1 significantly augments TGF-β1-induced lung FMT.

### YBX1 knockdown suppresses TGF-β1-mediated lung FMT

**Figure 3:**
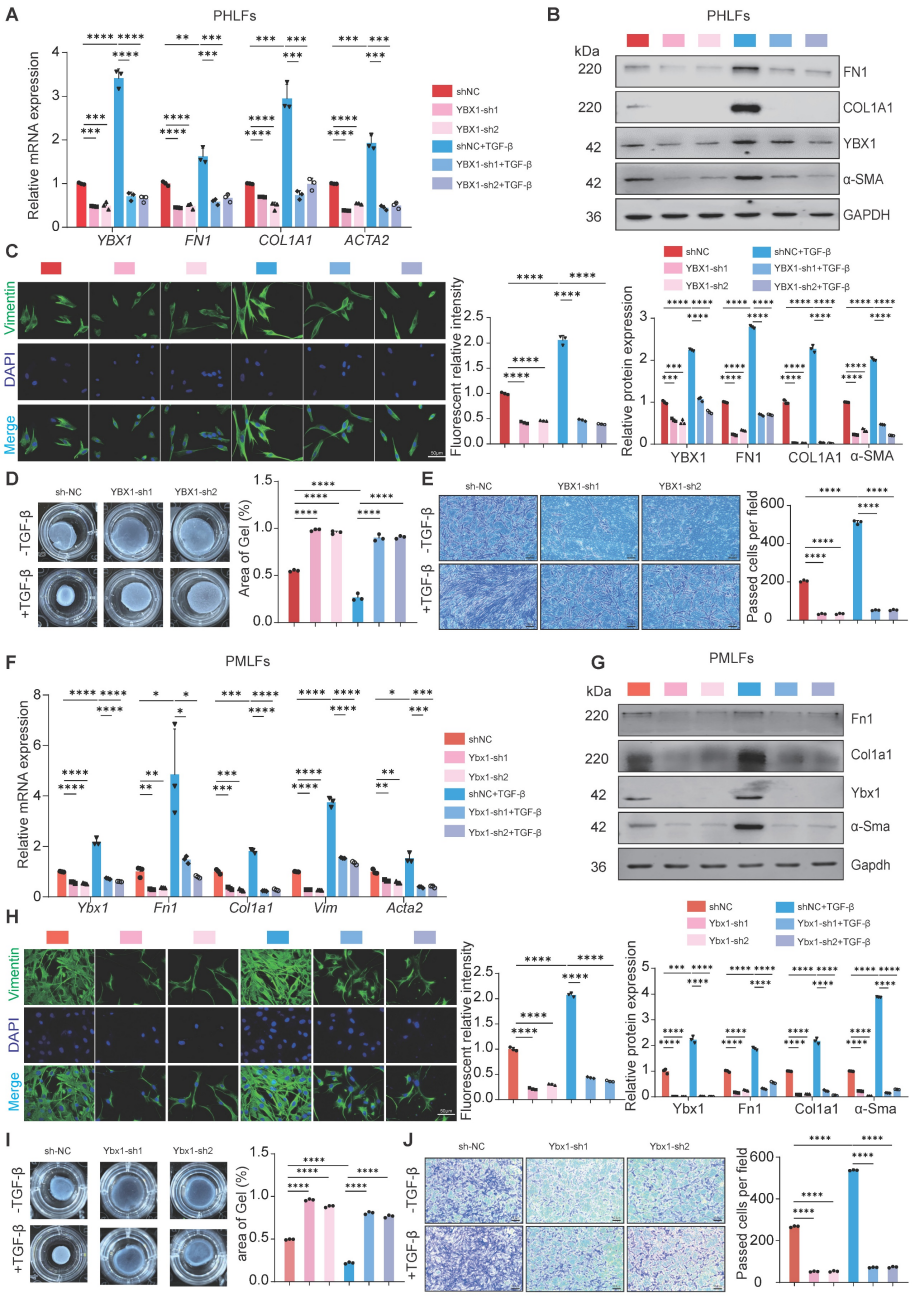
** YBX1 knockdown suppresses TGF-β1-mediated fibroblast activation in PHLFs and PMLFs. A**-**B** qRT-PCR(**A**) and Western blot(**B**) analysis of the expression of FN1, COL1A1 and α-SMA induced by TGF-β1 after YBX1 knockdown (n=3). **C** Immunofluorescence analysis revealed that YBX1 knockdown significantly inhibited the expression of vimentin in PHLFs. The nuclei were counterstained with DAPI. Bar = 20 μm, (n=3). **D** Transwell demonstrated the effect of YBX1 knockdown on PHLFs migration after 36 h. Scale bar = 100 μm, (n=3). **E** The fibroblast-induced CGC assay demonstrated that YBX1 knockdown in PHLFs inhibited fibroblast contractility (n=3). **F** qRT-PCR analysis validated the mRNA levels of *Ybx1, Fn1, Col1a1* and *Acta2* in PMLFs. Bars represent mean values of mRNA levels normalized to *Gapdh* mRNA (n=3). **G** Western blot assay showing the protein level of Fn1, Col1a1, Ybx1, α-Sma and Gapdh in PMLFs (n=3). **H** Immunofluorescence analysis revealed that Ybx1 knockdown significantly suppressed the expression of vimentin in PMLFs. The nuclei were counterstained with DAPI. Bar = 20 μm, (n=3). **I** The fibroblast-induced CGC assay demonstrated that Ybx1 knockdown in PMLFs inhibited fibroblast contractility (n=3). **J** Transwell demonstrated the effect of Ybx1 knockdown on PMLFs migration after 36 h. Scale bar = 100 μm, (n=3). Data are shown as the mean ± SD. Significance was assessed using 2-tailed Student's t-test. *P<0.05, **P<0.01, ***P<0.001, ****P<0.0001.

Next, to confirm the necessity of YBX1 for lung FMT, we knockdown YBX1 in PHLFs and PMLFs and treated them with TGF-β1 (10 ng/ml). Compared with the control group, YBX1 knockdown significantly suppressed TGF-β1-induced FN1, COL1A1 and α-SMA mRNA and protein expression in PHLFs (Fig. 3A, B). Consistent with these findings, immunofluorescence analysis revealed that YBX1 knockdown significantly suppressed the increase in the fluorescence intensity of TGF-β1-induced vimentin (Fig. 3C). Knockdown YBX1 expression significantly attenuated TGF-β1-mediated CGC capacity (Fig. 3D) and cell migration (Fig. 3E) in PHLFs. Similarly, Ybx1 knockdown in PMLFs significantly attenuated TGF-β1-induced FMT (Fig. 3F, J). Collectively, these findings suggest that YBX1 knockdown attenuates the differentiation of fibroblasts into myofibroblasts and inhibits TGF-β1-induced lung FMT.

### YBX1 binds and regulates PTBP1 to facilitate lung FMT

**Figure 4:**
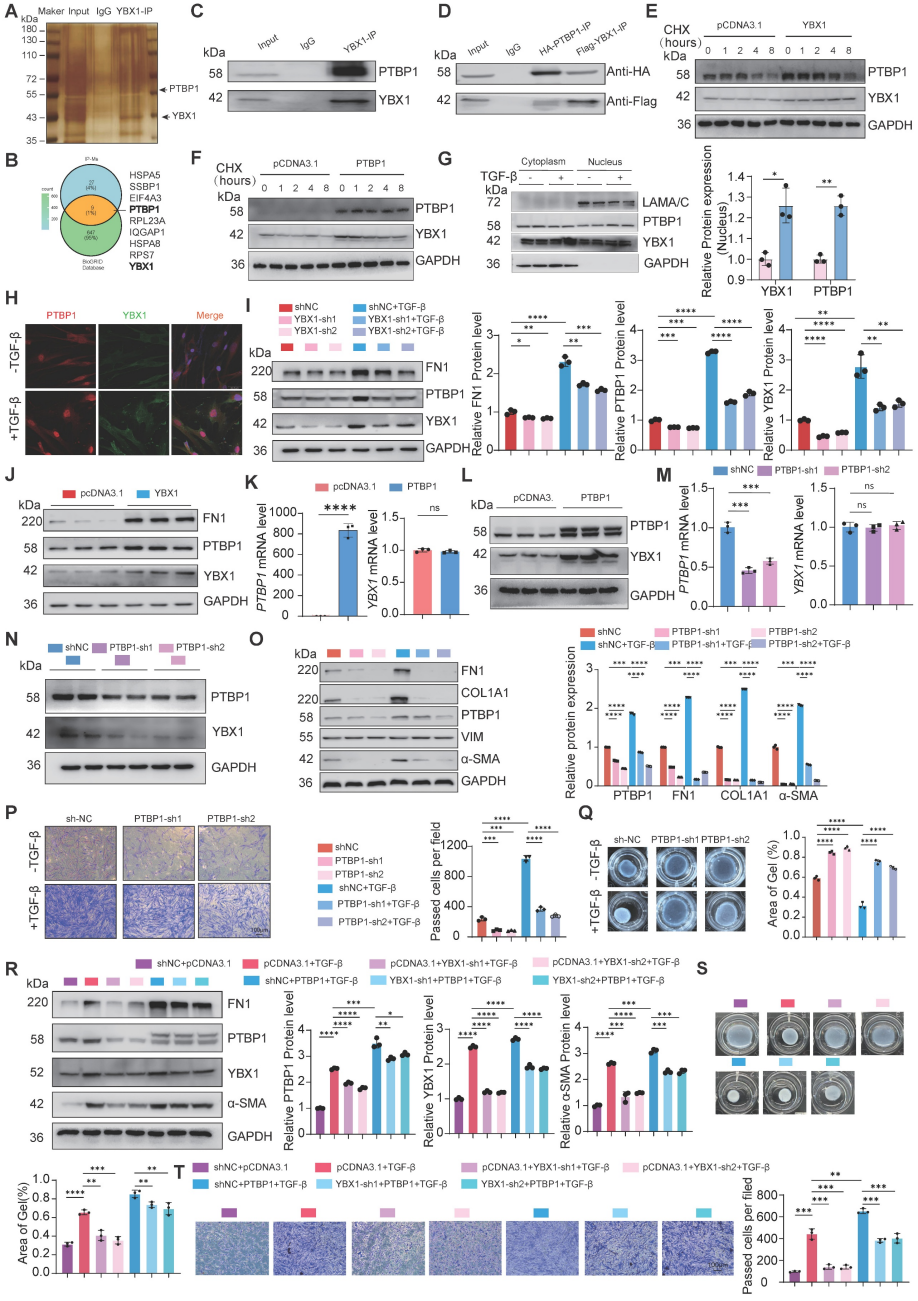
** YBX1 interacts with PTBP1 and regulates its expression. A** The silver staining assay to detect the IP results. **B** IP-MS and Venn diagrams was used to identify proteins that interact with YBX1. **C** Immunoprecipitation experiments were performed to analyze the interaction between YBX1 and PTBP1 in PHLFs. **D** Co-immunoprecipitation experiments analyzed the directly interaction between YBX1 and PTBP1 in 293T cells co-transfected with Flag-YBX1 and HA-PTBP1 plasmids. **E, F** Western blot to detect YBX1 and PTBP1 stabilized after overexpression each other (n=3). **G** TGF-β1 treatment increased the nuclear localization of YBX1 and PTBP1 in PHLFs (n=3). **H** Immunofluorescence analysis revealed that TGF-β1 treatment significantly increased the nuclear level of YBX1 and PTBP1 in PHLFs. Bar = 20 μm. **I** Knockdown of YBX1 reduced the protein expression levels of FN1, YBX1 and PTBP1 in PHLFs (n=3).** J** Overexpression of YBX1 in PHLFs increased the protein expression levels of FN1, YBX1 and PTBP1 (n=3). **K, L** qRT-PCR(K) and Western blot(L) were used to analyze the expression of YBX1 after PTBP1 overexpression (n=3). **M, N** qRT-PCR(M) and Western blot(N) were used to analyze the expression of YBX1 after PTBP1 knockdown (n=3). **O** Western blot analysis showed that the knockdown of PTBP1 in PHLFs inhibited the TGF-β1-induced protein expression of the fibrotic markers (n=3). **P** Transwell assay the effect of PTBP1 knockdown on PHLFs migration after 36 h (n=3). **Q** The CGC assay demonstrated that PTBP1 knockdown in PHLFs inhibited fibroblast contractility (n=3). **R** Western blot analyzes the expression of FN1, COL1A1, PTBP1, YBX1, and α-SMA induced by TGF-β1(10ng/ml) after YBX1 knockdown and PTBP1 overexpression (n=3). **S** CGC assay showing that PTBP1 overexpression could reverse the inhibitory effect of YBX1 knockdown on fibroblast activation (n=3). **T** Transwell assay showing that PTBP1 overexpression could reverse the inhibitory effect of YBX1 knockdown on PHLFs migration. Scale bar = 100 μm, (n=3). Data are shown as the mean ± SD. Significance was assessed using 2-tailed Student's t-test. *P<0.05, **P<0.01, ***P<0.001, ****P<0.0001.

To investigate the molecular mechanism by which YBX1 promotes lung FMT, we performed silver staining and immunoprecipitation-mass spectrometry on PHLFs and determined that PTBP1, which plays a role in various diseases and acts as a pro-fibrotic factor in fibrotic diseases 18, 19. Combined with BIOGRID database demonstrated that PTBP1 was the target of YBX1 in PHLFs (Fig. 4A, B). Coimmunoprecipitation and western blotting validated the interaction between YBX1 and PTBP1 (Fig. 4C, D). CHX chase assays showed that the half-life of both YBX1 and PTBP1 was significantly prolonged when the other partner was overexpression (Fig. 4E, F). Both YBX1 and PTBP1 significant increase in the activated PHLFs induced by TGF-β1 (Fig. 4G). Immunofluorescence analysis revealed that the colocalization of YBX1 and PTBP1 in the nucleus significantly increased after treatment with TGF-β1 (Fig. 4H). Knockdown of YBX1 significantly suppressed PTBP1 expression (Fig. 4I), whereas its overexpression increased PTBP1 levels (Fig. 4J). Parallelly, we examined the effects of overexpressing and knockdown PTBP1 on the expression of YBX1 at both the mRNA and protein levels. The results demonstrated that the overexpression of PTBP1 did not significantly affect the *YBX1* mRNA level but promoted YBX1 protein expression (Fig. 4K, L). While PTBP1 knockdown consistently inhibited YBX1 protein expression (Fig. 4M, N). Next, knockdown of PTBP1 attenuated the increases in FN1, COL1A1, and α-SMA levels mediated by TGF-β1 without affecting vimentin (Fig. 4O) but potently inhibited both TGF-β1-induced cell migration (Fig. 4P) and CGC capacity (Fig. 4Q) in PHLFs.

To further demonstrate whether YBX1 regulates the expression of PTBP1 to promote lung FMT, rescue experiments were performed, and the results revealed that overexpression of PTBP1 reversed the YBX1 knockdown induced reduction in FN1, and α-SMA expression (Fig. 4R) as well as the impairments in CGC capacity (Fig. 4S) and cell migration (Fig. 4T). Collectively, our results establish that YBX1 promotes TGF-β1-driven lung FMT through the direct binding and regulation of PTBP1.

### The profibrotic role of YBX1-PTBP1 requires PTPN1 expression

**Figure 5:**
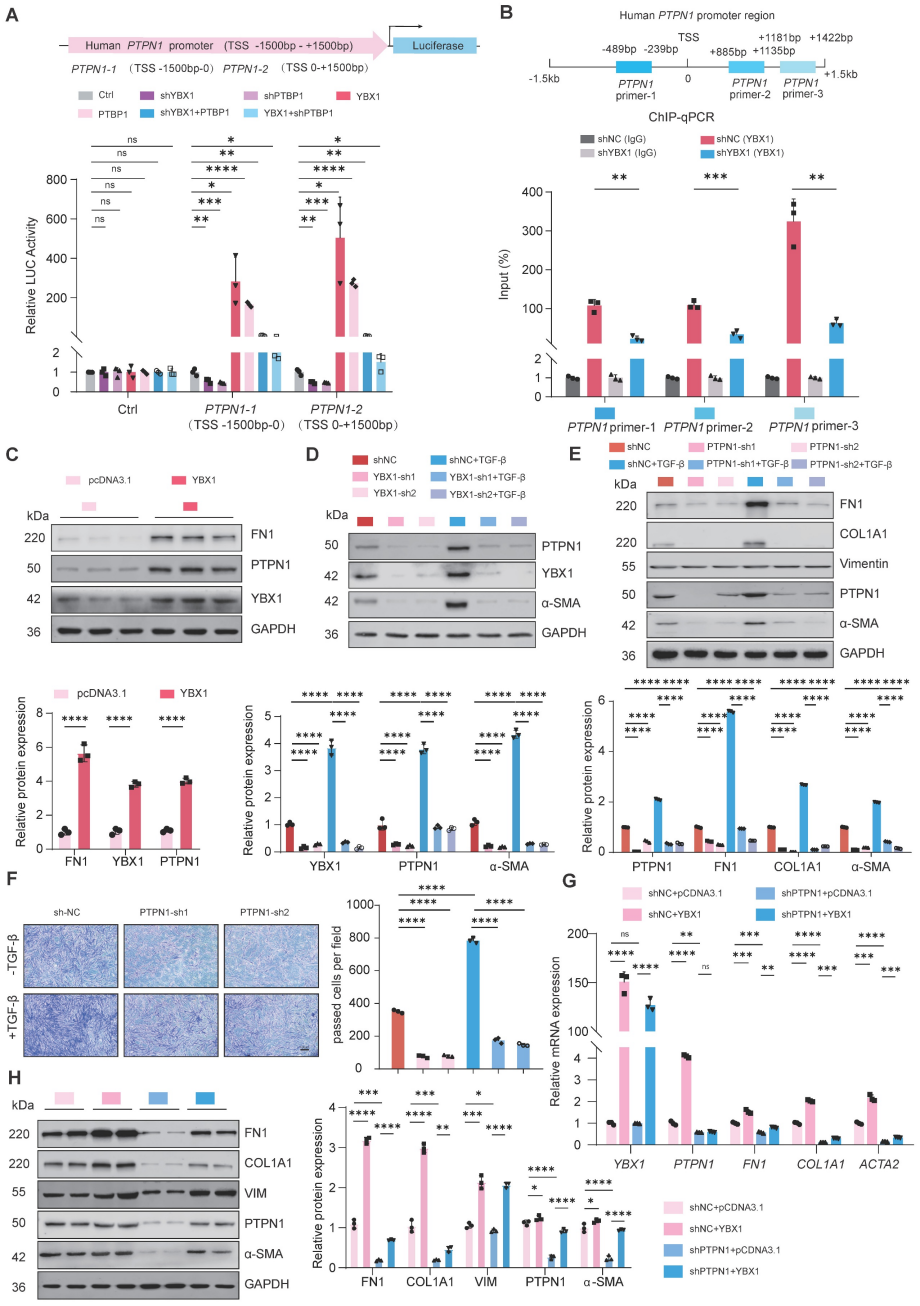
** YBX1-PTBP1 promotes the activation of fibroblasts by activating PTPN1. A** Dual-luciferase reporter assay revealed that knockdown of YBX1 or PTBP1 significantly reduced the luciferase activity of PTPN1 promoter, whereas their overexpression significantly increased it. Overexpression of YBX1 rescued the effect of PTBP1 knockdown on luciferase activity, and conversely, overexpression of PTBP1 rescued the effect of YBX1 knockdown on luciferase activity (n=3). **B** ChIP-qPCR analysis showed that YBX1 significantly binds to the promoter region of PTPN1 (n=3). **C** Overexpression of YBX1 in PHLFs increased the protein expression levels of fibronectin and PTPN1 (n=3). **D** Knockdown of YBX1 in primary human lung fibroblasts reduced the protein expression levels of α-smooth muscle actin and PTPP1 (n=3). **E** Western blot analysis showed that knockdown of PTPN1 in PHLFs inhibited the expression of fibronectin, collagen type I, and α-smooth muscle actin induced by TGF-β1 (n=3). **F** Transwell assay analyzed the inhibition of TGF-β1-induced invasiveness in primary human lung fibroblasts after PTPN1 knockdown (n=3). **G, H** qRT-PCR (G) and Western blot (H) analyses showing that knocking down PTPN1 reverse the excessive deposition of extracellular matrix components such as collagen induced by YBX1 overexpression (n=3). Data are shown as the mean ± SD. Significance was assessed using 2-tailed Student's t-test. *P<0.05, **P<0.01, ***P<0.001, ****P<0.0001.

To further investigate the potential targets of YBX1 and PTBP1, YBX1 interact with PTBP1 which is involved in fibrosis progress, we hypothesized that YBX1 and PTBP1, both implicated in fibrosis, might coregulate through PTPN1. Next, we constructed luciferase reporter vectors containing the *PTPN1-1* (TSS -1.5k-0 bp) and *PTPN1-2* (TSS 0-1.5k bp) promoter regions. Dual-luciferase reporter assays revealed that knockdown of either YBX1 or PTBP1 expression significantly suppressed the luciferase activity driven by the *PTPN1* promoter, and this suppression induced by YBX1 knockdown was reversed by PTBP1 overexpression (Fig. 5A). Chromatin immunoprecipitation followed by quantitative PCR (ChIP-qPCR) revealed that YBX1 binds to the *PTPN1* promoter region, and YBX1 knockdown substantially decreased this occupancy, leading to significantly reduced *PTPN1* transcription (Fig. 5B). Consistent with these findings, western blot analyses revealed that YBX1 overexpression or knockdown potently increased or suppressed the expression of PTPN1, respectively (Fig. 5C, D). Moreover, PTPN1 deficiency potently suppressed both the increase in the expression of profibrotic genes and the increase in TGF-β1-driven cell migration (Fig. 5E, F). Furthermore, WB and qRT-PCR revealed that YBX1 overexpression reversed the PTPN1 deficiency-induced attenuation of profibrotic gene expression and ECM deposition (Fig. 5G, H). Together, YBX1 interacts with PTBP1 to regulate the PTPN1 expression and promoting lung FMT.

### AAV-mediated delivery of Ybx1 shRNA alleviates BLM-induced lung fibrosis

**Figure 6:**
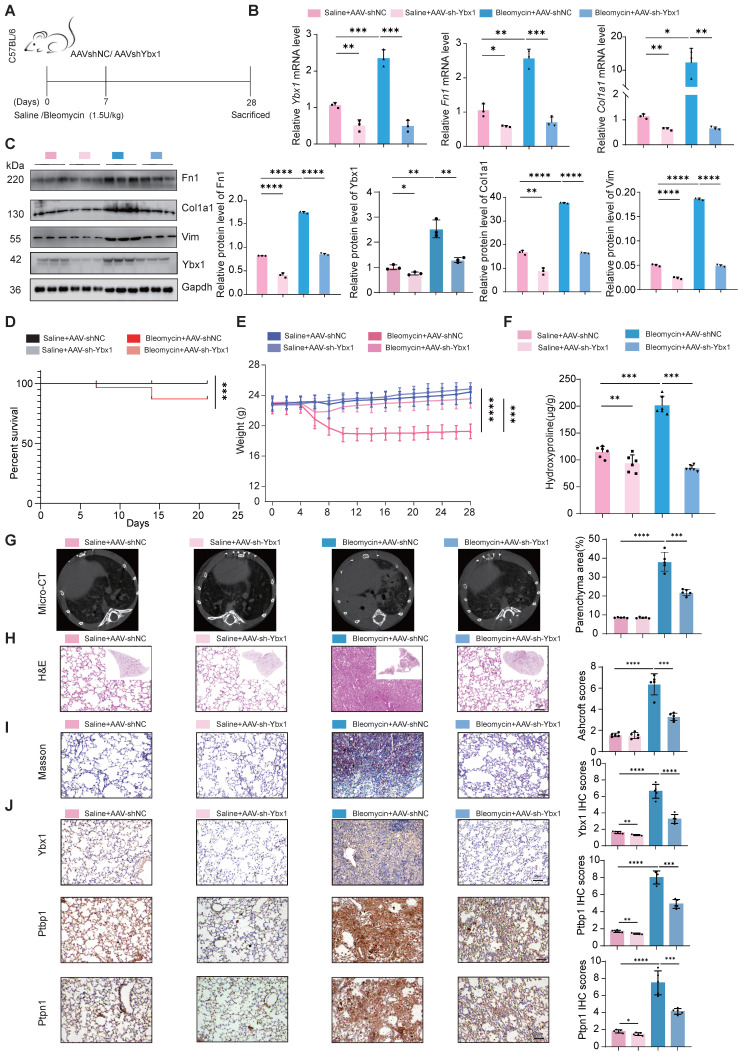
** Ybx1 Knockdown significantly ameliorated BLM-induced pulmonary fibrosis in mice. A** Schematic diagram describing the experimental procedure in a saline or BLM-induced pulmonary fibrosis mouse model, with intratracheal instillation of AAV-Ybx1 shRNA or AAV-NC in the mouse model. **B** qRT-PCR analysis of the transcriptional changes of *Ybx1, Fn1* and *Col1a1* in lung tissue of mice treated with saline or BLM and treated with AAV-Ybx1 shRNA or AAV-NC (n=3). **C** Western blot analysis of the protein level of Ybx1, Fn1, Col1a1 in lung tissue of mice treated with saline or BLM and treated with AAV-Ybx1 shRNA or AAV-NC (n=3). **D** Percent survival of mice treated with saline or BLM and treated with AAV-Ybx1 shRNA or AAV-NC (n =10).** E** Body weight curves of mice treated with saline or BLM and treated with AAV-Ybx1 shRNA or AAV-NC (n=10). **F** Hydroxyproline content analysis of the entire right lung of mice treated with saline or BLM and treated with AAV-Ybx1 shRNA or AAV-NC (n=3). **G** Representative micro-CT images and quantitative analysis of fibrotic areas in mouse lungs (n=5). **H** Representative microscopic images of H&E staining. **I** Representative microscopic images of Masson's trichrome staining and quantitative analysis of fibrotic areas in mouse lungs (n =5). **J** Ybx1, Ptbp11 and Ptpn1 IHC staining on lung tissue sections from each group of mice (n=5). Data are shown as the mean ± SD. Significance was assessed using 2-tailed Student's t-test. *P<0.05, **P<0.01, ***P<0.001, ****P<0.0001.

To validate our findings *in vivo*, we constructed a mouse model of lung fibrosis by intratracheal instillation of BLM. Seven days post-injection, we treated the mice with adenovirus-associated virus (AAV)-Ybx1-shRNA (Fig. 6A). qRT-PCR and western blot analyses revealed that Ybx1 knockdown significantly reduced BLM-induced expression of profibrotic markers, including Fn1 and Col1a1, at both the transcriptional and protein levels (Fig. 6B, C). Survival analysis revealed that the survival rate of mice in the pulmonary fibrosis model group was significantly lower than that in the AAV-Ybx1-shRNA group (Fig. 6D).

Compared with the control group, Ybx1 knockdown significantly ameliorated disease-associated weight loss (Fig. 6E) and reduced the hydroxyproline concentration (Fig. 6F). In addition, micro-CT imaging revealed that extensively dense material infiltration and increased opacity in the lungs of BLM-treated mice were markedly reduced by Ybx1 knockdown (Fig. 6G), as shown by H&E staining, Masson's trichrome staining, and immunohistochemical (IHC) staining. Ybx1 knockdown significantly attenuated the structural destruction, collagen accumulation and the expression of Ptbp1 and Ptpn1 caused by BLM (Fig. 6H-J). Taken together, these data indicate that Ybx1 knockdown can effectively attenuate susceptibility to pulmonary fibrosis *in vivo*.

## Discussion

Our research consistently demonstrated that YBX1 is upregulated in the lung of patients with IPF and in the BLM mouse model of pulmonary fibrosis. *In vitro*, overexpression of YBX1 promoted lung FMT, whereas in TGF-β1-treated PHLFs, YBX1 markedly increased the invasiveness and contractility of primary lung fibroblasts. Conversely, the downregulation of YBX1 expression reduced the expression of FN1, COL1A1, and α-SMA induced by TGF-β1 and suppressed fibroblast invasion and contraction. These results indicate that YBX1 plays a pro-fibrotic role in lung FMT. Importantly, the therapeutic relevance of these findings was confirmed *in vivo*, as the administration of AAV-shYbx1 to the BLM-induced mouse model of pulmonary fibrosis significantly attenuated collagen deposition and improved lung architecture. These results strongly suggest that targeting YBX1 could be a viable therapeutic strategy for the treatment of pulmonary fibrosis.

IPF is a progressive interstitial lung disease characterized by lung injury that activates fibroblasts and drives their differentiation into myofibroblasts 5. This process leads to the formation of fibroblast foci and the excessive deposition of ECM components such as collagen, resulting in pulmonary scarring and dysfunction and ultimately respiratory distress and death 20-22. YBX1 is a multifunctional RNA-binding protein involved in various cellular processes, including transcriptional regulation, mRNA stability, and translational control 23. YBX1 plays an oncogenic role in various cancers and is a profibrotic driver of fibrosis 24, 25. However, its role in the pathogenesis of pulmonary fibrosis has remained unknown. This study identifies YBX1 as a critical regulator of fibroblast activation in the lung and elucidates a novel signaling pathway through which it exerts its profibrotic effects.

To elucidate the mechanism underlying the effects of YBX1 on pulmonary fibrosis, we investigated potential molecular partners of YBX1 and confirmed the direct interaction of YBX1 with PTBP1. PTBP1 is another important RNA-binding protein involved in regulating RNA splicing, stability, and translation 26-30. Consistent with these findings, our study demonstrated that PTBP1 knockdown inhibited TGF-β1-induced fibroblast activation and ECM deposition in lung fibroblasts. We further elucidated that YBX1 positively regulates PTBP1 expression, suggesting a hierarchical relationship in which YBX1 acts upstream to control PTBP1 levels. The functional importance of this interaction was highlighted by our finding that the profibrotic effects of YBX1 were dependent on PTBP1, indicating that PTBP1 is a key downstream mediator of YBX1-driven fibroblast activation. Our findings indicate that the YBX1-PTBP1 complex promotes the stabilization of both proteins. Consequently, the stabilized complex appears to exhibit enhancement of downstream gene transcriptional activity relative to either protein alone. This functional enhancement may be attributable to increased nuclear retention and/or the recruitment of distinct co-activators that neither YBX1 nor PTBP1 can efficiently recruit independently.

PTPN1, a protein tyrosine phosphatase, plays a crucial role in cell signaling, primarily by dephosphorylating tyrosine residues to regulate various cellular functions, including proliferation, migration, and apoptosis 31-33. Our study revealed that YBX1 binds to the promoter region of *PTPN1* to regulate its expression. Additionally, PTPN1 knockdown inhibited the differentiation of fibroblasts into myofibroblasts and the deposition of ECM.

These results suggest that PTPN1 acts as a downstream target of YBX1 in the pathogenesis of pulmonary fibrosis. Our study provides insight into the role of the YBX1/PTBP1/PTPN1 axis in pulmonary fibrosis; however, the specific mechanisms by which YBX1-PTBP1 complex to regulate PTPN1 expression require further in-depth investigation, the upregulation of PTPN1 by this complex may involve mediating its transcriptional activation or affecting post-transcriptional regulatory processes. Moreover, our findings highlight the potential synergistic effects of YBX1 and PTBP1 in pulmonary fibrosis. Their combined action likely promotes fibrosis by dysregulating intracellular signaling and enhancing cell proliferation and migration. This collaborative mechanism warrants further exploration to better understand the complexity of pulmonary fibrosis.

This study has several limitations. First, although our research focused primarily on fibroblasts, the potential role of YBX1 in other cell types within the pulmonary fibrosis microenvironment remains unexplored. Second, we employed a commonly used AAV delivery approach for *in vivo* knockdown, rather than utilizing fibroblast-specific knockout mouse models, which would provide more targeted validation of the cell-autonomous functions of YBX1. Furthermore, the BLM-induced mouse model of pulmonary fibrosis, although widely utilized, does not fully recapitulate the complex and chronic pathogenesis of IPF. Therefore, the therapeutic potential of YBX1 as a drug target for IPF requires further validation in more clinically relevant models.

In summary, our study revealed a previously unknown role for the RNA-binding protein YBX1 in pulmonary fibrosis. We elucidated a novel signaling pathway in which YBX1 interacts with and upregulates PTBP1. This complex then cooperatively drives the expression of PTPN1, leading to the activation of lung fibroblasts during the progression of pulmonary fibrosis. Our *in vivo* experiments indicate that inhibition of YBX1 can ameliorate established pulmonary fibrosis in mice. These findings not only provide new insights into the molecular mechanisms underlying pulmonary fibrosis but also validate the YBX1/PTBP1/PTPN1 axis as a promising new set of therapeutic targets for this devastating disease.

## Materials and Methods

### Lung tissue sampling

Lung tissue samples used for immunohistochemical staining and the isolation of primary lung fibroblasts were taken from the Henan Provincial Chest Hospital. IPF samples were surgical remnants of lungs from IPF patients undergoing lung transplantation. Control lung tissue samples were obtained from normal histological tissue at the disease-free margins of resected lung cancer specimens. All participants provided written informed consent, and the study protocol was approved by the Human Ethics Committee of Henan Chest Hospital (Approval No. 2022-052).

### Mouse model

Animal care and handling procedures were conducted in accordance with the guidelines established by the Institutional Animal Care and Use Committee (IACUC SMKX-2118BS1018) of Henan Normal University, adhering to the standards set by the Animal Behavior Society and national regulations. Male C57BL/6N mice, 8 to 10 weeks old, 18 to 22 gram were purchased from Charles River Laboratories (Beijing, China) and housed in a specific pathogen-free environment. After a 7-day acclimation period, the mice were lightly anesthetized and intratracheally injected with a single dose of 50μl of either saline or 1.5 U/kg BLM (Nippon Kayaku Co., Tokyo, Japan) dissolved in PBS. One week after the intratracheal instillation of BLM, mice were administered with 50μL of pAAV-CMV-NC or pAAV-CMV-Ybx1-shRNA (target sequence, GAGAACCCTAAACCACAAGAT, viral titer: 1.0 × 10^13^ vg/mL) via intratracheal instillation.

### Micro-CT imaging

Quantitative measurement of mouse lung tissue density was performed using CT to determine the degree of pulmonary fibrosis in each group. CT scanning was conducted as follows: after anesthesia with isoflurane, mice were placed in a supine position on the scanning bed, and their chests were secured with tape. After setting the scanning parameters, in vivo lung tissue was scanned. Images were automatically generated by the system workstation and post-processed with relevant software. Structural and morphological changes in lung tissue were observed and compared with the control group to assess varying degrees of pulmonary fibrosis.

### Hydroxyproline assay

The content of pulmonary hydroxyproline was measured using a hydroxyproline assay kit (Sigma-Aldrich, USA) 34, with the experimental procedure conducted according to the manufacturer's instructions. The data were calculated and expressed as μg of hydroxyproline per gram of lung tissue.

### H&E and Masson's trichrome staining

Dewaxing and rehydrating the lung tissue, hematoxylin and eosin (H&E) staining 35 and Masson's trichrome staining (Solarbio, Beijing, China) were performed according to the kit instructions, and tissue histopathological changes were observed under the microscope.

### IHC

IHC staining was performed on the lungs of IPF and BLM-treated mice. The sections were then blocked with 5% goat serum for 30 minutes and incubated with a primary antibody against YBX1 at 4 °C overnight. The next day, after washing with PBS, the sections were incubated with a horseradish peroxidase-conjugated secondary antibody at 37°C for 30 minutes, followed by development with a DAB chromogen. Nuclei were counterstained with hematoxylin for 5 minutes, after which the sections were washed clean with PBS. After sealing with a neutral resin, the stained lung sections were photographed using an optical microscope.

### Cell culture and treatment

The 293T cells were purchased from the American Type Culture Collection (ATCC). PHLFs, PMLFs and 293T cells were cultured in DMEM (containing 10% fetal bovine serum, 100 U/mL penicillin, and 100 µg/L streptomycin) in a cell culture incubator at 37 °C and 5% CO2. The culture medium was replaced every three days. PHLFs or PMLFs were treated with TGF-β1 (PeproTech, #100-21C, USA) at a final concentration of 10 ng/ml. Following a 24-hour incubation, samples were collected for subsequent analysis.

### The isolation of primary lung fibroblasts

After collecting lung tissue from patients or mouse, the tissue was washed twice with sterile PBS, and then dissected into small pieces of about 1mm³. Collagenase (1mg/mL) was added, and the tissue was digested for 30 minutes at 37 °C. Subsequently, the cells were centrifuged at 500g for 5 minutes, washed, and resuspended in complete medium, which consisted of DMEM containing 10% fetal bovine serum, 100U/mL penicillin, and 100 µg/L streptomycin (Solarbio). The cell suspension was then evenly plated into a 10cm cell culture dish and cultured in a cell culture incubator at 37 °C and 5% CO2. After 4-5 days of culture, primary lung fibroblasts were obtained for subculture or other assays. The cells used exhibited the typical spindle-shaped, elongated morphology characteristic of fibroblasts. The identity of the fibroblasts was confirmed by positive immunofluorescence staining for α-SMA. All experiments utilized cells between passages 3 and 8.

### qRT-PCR and Western blot analysis

Total RNA was extracted using Trizol reagent 36, cDNA was synthesized using a reverse transcription kit (YEASEN, Shanghai, China), and the concentration and purity of the cDNA were analyzed using a Nanodrop spectrophotometer. Subsequently, qRT-PCR was performed under the Light Cycler 96 fluorescence quantitative PCR system (Roche) using a SYBR Green kit (YEASEN, Shanghai, China) according to the manufacturer's instructions. Data were calculated using the 2-∆∆Ct method. The qRT-PCR primers used in this study are as follows.

For western blot, the following antibodies were used: YBX1 (20339-1-AP, Proteintech), GAPDH (AF7021, Affinity), α-SMA (ab124964, Abcam), Type I collagen (14695-1-AP, Proteintech), Fibronectin (15613-1-AP, Proteintech), Vimentin (10366-1-AP, Proteintech).

### Transwell assay

Treated primary lung fibroblasts were digested from the culture vessel, the cells were resuspended in serum-free DMEM and counted. Then, 600μL of DMEM medium containing 10% fetal bovine serum was added to a 24-well plate, and a Transwell insert(8µm) was placed into the well. Subsequently, an equal number of cells were seeded on the upper chamber of the Transwell in the 24-well plate and cultured in a cell culture incubator at 37°C and 5% CO2 for 24 hours. After 24 hours, the transwell inserts were washed with PBS buffer and fixed with 4% paraformaldehyde at room temperature for 30 minutes, stained with crystal violet solution for 10 minutes, and observed and imaged under a microscope.

### Collagen gel contraction assay

Primary lung fibroblasts were mixed with collagen at a specific ratio and seeded into a 24-well plate. The 24-well plate was then incubated in a cell culture incubator at 37 °C and 5% CO2 to observe the different degrees of gel contraction. Afterward, the contracted gel area was measured using ImageJ software.

### Immuno-fluorescence assay

Cells that have been treated are fixed at room temperature in 4% paraformaldehyde. After treating with 0.3% TritonX-100 for 5 minutes. The antibody was incubated overnight at 4 °C, followed by incubation with a specific secondary antibody. The cell nuclei were stained with DAPI.

### IP and Co-IP

The Co-IP and IP experiments were performed as previously described 37. 293T cells were co-transfected with two plasmids, and 48 hours later, the cells were lysed using Co-IP lysis buffer. After cell lysis, the samples were treated with an ultrasound cell disruptor, followed by centrifugation. Protein A/G Beads were then added, and the mixture was incubated overnight at 4 °C. The interacting complexes obtained from the magnetic beads were subjected to Western blot analysis. Using a similar method, we performed IP experiments in primary lung fibroblasts.

### Silver staining

The SDS-PAGE gel was stained using a silver staining kit according to the manufacturer's instructions, following the Silver Quest Silver Staining Kit (Thermo, USA).

### ChIP-qPCR assay

The ChIP-qPCR assay was performed as previously described 38. Primary lung fibroblasts were digested and washed with PBS buffer, followed by crosslinking with 1% formaldehyde for 10 minutes at room temperature. Crosslinking was quenched with 125 mM glycine for 5 minutes The chromatin was incubated with YBX1 antibody overnight at 4 °C. Anti- IgG was used as a negative control. The enriched DNA fragments were then purified. Subsequently, qRT-PCR analysis was performed using a SYBR Green kit (YEASEN, Shanghai, China).

### Dual luciferase reporter gene assay

The Dual Luciferase Reporter Gene Assay was performed using the Dual Luciferase Reporter Gene Assay Kit (YEASEN, Shanghai, China) to measure the activity of luciferase. The short sequences *PTPN1*-3'UTR-1 and *PTPN1*-3'UTR-2 were inserted into the pGL3.0 luciferase reporter gene vector. Subsequently, 293T cells were seeded in a 24-well plate, and plasmids including TK, pGL3.0, sh-NC, pcDNA3.1, shYBX1, shPTBP1, YBX1-OE, and PTBP1-OE were transfected into the seeded 293T cells. After 48 hours, the samples were collected and the Dual Luciferase Reporter Gene Assay Kit was used to determine the results.

### Statistical analysis

Experimental data from this study were analyzed using GraphPad Prism 8 (GraphPad Software, Inc., San Diego, CA, USA), with at least three independent replicates per group. Two-tailed Student's t-tests were applied to assess the statistical significance of differences between groups. Data are expressed as mean ± standard deviation (SD), and a p-value < 0.05 was considered statistically significant.
